# Improvement in the quality and productivity of *Codonopsis pilosula* seedlings by dazomet soil fumigation

**DOI:** 10.1038/s41598-024-56093-3

**Published:** 2024-03-05

**Authors:** Hongyan Wang, Yuan Chen, Fengxia Guo, Pengbin Dong, Wei Liang, Jiali Cheng

**Affiliations:** College of Agronomy, College of Life Science and Technology, State Key Laboratory of Aridland Crop Science, Gansu Agricultural University, Lanzhou, 730070 China

**Keywords:** *Codonopsis pilosula*, Dazomet, Quality, Seedlings, Yield, Physiology, Plant sciences

## Abstract

Dazomet is a dry powder formulation that releases toxic gas containing methyl isothiocyanate, which controls soil-borne pests and weeds, improving crop yields when applied to moist soils. To explore the efficacy of dazomet fumigation in the cultivation of the perennial herb *Codonopsis pilosula*, four typical cultivars (G1, G2, W1 and TCK) in Gansu Province were selected for seedling cultivation after soil fumigation (F) by dazomet, and non-fumigated soil was used as a control (CK). The experiments took 2 years to complete. The functional diversity of the soil enzymes and microorganisms, seedling emergence and physiological characteristics, and the quality and yield of *Codonopsis* seedlings and Radix were assessed. The results showed that the seed emergence rate, seedling re-green rate and several antioxidant enzymatic activities improved in the treatments involving soil fumigation with dazomet, and membrane lipid peroxidation in the seedlings decreased. On average, compared with those of the respective controls, the root viability and yield of the seedlings of the tested cultivars also increased by 34.87% and 42.4%, respectively, and the incidence of root rot in the seedlings was reduced by 83.9%, compared with their respective controls. After harvest, the yield increased by 23.9%, the incidence of root rot decreased by 61.3%, increase in yield and a 61.3% reduction in incidence, and the medicinal materials were determined to be safe and residue-free. The effects of fumigation were cultivar-specific and were especially prominent in G2. Therefore, soil fumigation with dazomet could improve the quality and productivity of *Codonopsis pilosula* seedlings. Taken together, these findings suggest that when herbs are bred by seedling transplantation, especially cultivars of good quality but poor resistance or species with rare germplasm resources, soil fumigation provides a way to improve cultivation effectiveness and, more importantly, ensures the probability of successfully breeding the species.

## Introduction

*Codonopsis pilosula* is a traditional herbal plant that is widely used in Asian countries, mainly planted in China, Japan, and Korea^1,2^, and its dry roots are used as medicine (*Codonopsis* Radix, also Dangshen)^3^. The use of this herb has been extensively recorded in Traditional Chinese medicine (CHM) texts, such as “Ben Cao Cong Xin”, “Supplements to Compendium of Materia Medica” and “Ben Cao Qiu Zhen”^4^. Furthermore, more than 110 CHM preparations containing *Codonopsis* Radix or its extracts are listed in the Chinese Pharmacopoeia^3^. *Codonopsis* Radix is also widely used as a food additive in wine, soup, porridge, etc.^5,6^. Moreover, these agents have a wide range of pharmacological effects, such as strengthening the spleen, benefiting the lungs, nourishing the blood, and promoting the production of body fluids^3,5,7,8^. People in countries such as China, Japan, North Korea, South Korea, and the United States also use it as a food ingredient^9,10^. Owing to these health benefits, the demand for *Codonopsis* Radix has increased globally, stimulating the large-scale cultivation of *C. pilosula*, especially in Gansu Province^11,12^. Gansu Province has more than 90% of the *C. pilosula* plantation area^13^ in China and was recognized as “The Hometown of *C. pilosula* in China” by the Chinese Special Products Township Committee in 2001. Because the medicinal quality of these plants is closely related to their origin, the plantation area of *C. pilosula* in Gansu Province is limited. Continuous planting has caused a decrease in plant quality and yield. Thus, studies are urgently needed to overcome these continuous cropping obstacles and improve medicinal quality and productivity.

It has been reported that exogenous soil, intercropping^14^, crop rotation^15^, the application of organic materials^16^, and soil fumigation^17^ can reduce soil-borne diseases, pests, and weeds. Soil fumigation is the most effective and stable method for protecting crops from soil-borne diseases, nematodes, and weeds during continuous cropping^18,19^. Dazomet (DZ) is a highly effective microbicide for controlling fungi, weeds, and subterranean pests^20–22^; it induces a carbonylation reaction at nucleophilic sites (such as amino, hydroxyl, and thiol groups). Most studies of DZ have been performed on strawberry, tomato, flower, ginger, cucumber, and other high-value crops^23–26^. It can also be applied in integrated pest management programs for ginger to control ginger bacterial wilt in China^24^, and it has successfully replaced methyl bromide, which damages the ozone layer^27^.

*Codonopsis pilosula* is a perennial herbaceous plant in the Campanulaceae family that mainly propagates by seed reproduction^28^. The yield and quality of *Codonopsis* Radix depend on the *C. pilosula* seed and seedling quality. High-quality *Codonopsis* seedlings are fundamental for improving the *Codonopsis* Radix yield and quality^29,30^. However, several problems have limited the quality and production performance of *C. pilosula* during cultivation. First, the seeds of *C. pilosula* are tiny and the weight of 1000 seeds is only approximately 0.30 g, which limits the emergence rate. Second, the occurrence of various weeds strongly affects the seedling growth and development. Third, in the native producing areas, growers have implemented continuous cropping instead of crop rotation to pursue high profits, which can lead to soil-borne diseases, pests, and weeds, strongly affecting *Codonopsis* seedling quality. Although many studies have been performed on evaluating the effects of sowing time, sowing density, seed treatment, cover materials and sowing methods^31^, these were aimed at increasing production and thus failed to solve problems related to quality. Therefore, the present study was designed to improve *Codonopsis* seedling quality through soil improvement. Briefly, four typical cultivars of *C. pilosula* in Gansu Province were selected for seedling cultivation after soil fumigation with DZ. The seed emergence rate, root viability, antioxidant enzymatic activity, transplant re-green rate, and other indicators of the plant seedlings in the fumigated and non-fumigated plots were recorded. Additionally, the quality and yield of the seedlings and Radix were measured, after which the safety of the medicine was evaluated. The present study was aimed at providing systematic findings to promote the sustainable and efficient cultivation of *C. pilosula* while also protecting the environment.

## Materials and methods

### Experimental field and test cultivars

The seedling cultivation experiment was conducted in Lichuan (E 104° 01ʹ, N 33° 46 ʹ) in northern Tanchang County, Longnan city, Gansu Province, China, which is the main production area of *C. pilosula* and has a mild and humid climate. The previous crop in the experimental field was *Codonopsis pilosula* (continuous cropping). To eliminate test errors, four cultivars, “Gandang No. 1” (G1) and “Gandang No. 2” (G2), bred by the Eco-cultivation and Breeding Group of Professors Yuan Chen and Fengxia Guo at Gansu Agricultural University, the local representative cultivar “Weidang No. 1” (W1) and the Tanchang traditional cultivar (TCK), were used synchronously in this experiment. After *Codonopsis* seedlings were harvested, transplanted in a *Scutellaria baicalensis* stubble field without any soil treatment in Pangjiacha (E 107° 87ʹ, N 44° 44ʹ) in Fuxing, Longxi County, Gansu Province.

### Experimental design

A two-factor (soil pretreatment and cultivar) scheme was used in the seedling cultivation experiment, in which the soil pretreatment factor included two levels of soil fumigation (F) by 98% dazomet and non-fumigation (CK). Dazomet particles (98%) were obtained from Jiangsu Nantong Chemical *Co*., *Ltd*.. Based on preliminary results from our laboratory pot experiments and the findings of other researchers with respect to other crops^20,25,32^, the applied concentration was set to 45 g m^−2^, and the duration of fumigation was 25 days. A paired design for soil fumigation and non-fumigation treatments was used for the four cultivars in this experiment, including eight experimental plots of 30 m^2^ (6 m × 5 m) in each block. To facilitate soil fumigation and field management, the experimental field was divided into two adjacent parallel long blocks from north to south, separated by ridges 0.80 m wide and 0.50 m high. According to the NY/T 3129-2017 technical specification for soil fumigation with dazomet^33^, the soil was fumigated in the northern long block with 45.0 g m^−2^ dazomet on Mar. 15, 2019, and the non-fumigation one in the southern region was used as the control. At the end of fumigation, soil fertility was measured. The results were consistent (nonsignificant difference according to *t*-test, *P* > 0.05) between the two blocks (local production fields Table 1). Then, each long block was divided into four plots separated by a 0.5 m ridge, in which the four cultivars were randomly sown to cultivate seedlings. Other than the different treatments applied, the field management and operation procedures used were the same as those used for local production fields.

**Table 1:** Soil basic fertility status in the seedling cultivation block of *Codonopsis pilosula.*

Soil treatments	Experiment blocks			
	F	CK	*t*	*P* value
Water content (%)	75.79 ± 0.61	76.78 ± 2.58	0.375	0.727
Organic carbon (g kg^−1^)	10.46 ± 0.29	9.70 ± 0.36	1.661	0.172
Organic matter (g kg^−1^)	18.04 ± 0.50	16.72 ± 0.61	1.661	0.172
Nitrate (mg kg^−1^)	25.66 ± 0.44	25.42 ± 0.87	0.251	0.814
Ammonium nitrogen (mg kg^−1^)	16.42 ± 0.57	17.13 ± 0.93	0.651	0.551
Olsen-P (mg kg^−1^)	15.15 ± 1.45	15.69 ± 0.75	0.341	0.750
Available potassium (mg kg^−1^)	128.75 ± 3.96	129.77 ± 5.36	0.153	0.886

F means soil fumigation, and CK means soil non-fumigation. The data in the table is the X ± SD.

### Seed sowing

Seeds of the four cultivars of *C. pilosula* were sown in the above plots of the F and CK blocks on 7 May 2019. The seeds were uniformly spread and were distributed by a single person, with a seed density of 3.0 g m^−2^ during sowing. After sowing, the block was covered with a black shading net (60% shading rate). The seedlings were ordinally sampled at three locations in each plot at three stages of seedling cultivation (18 Aug., 22 Sep., and 20 Oct.) and were subsequently transported to the laboratory. The sampled seedlings were washed briefly in running water to remove soil residues. Surface moisture was removed with absorbent paper, and the samples were subsequently stored at -80 °C for determination of physiological indicators.

### Determination of soil indicators

The soils were sampled by a multipoint method with a soil auger before sowing and at the middle and late stages of seedling growth and were mixed as composite soil samples of the 0–10 cm and 10–20 cm layers in each block. The samples were subsequently placed in a tagged aseptic self-sealing bag and transported to the laboratory to be stored in a refrigerator at − 80 °C for subsequent determination of soil microorganisms, functional diversity of the soil communities and enzymatic activity.

Soil enzymatic activity was determined according to methods by Guan et al*.*^34^. The quantity of culturable soil microbes was determined using a dilution plate coating method. Soil samples were accurately weighed to 10.0 g and dissolved in 90 mL of dd-water with shaking and mixing; after the samples were left undisturbed, the supernatant was aspirated and diluted 10^–3^, 10^–4^, 10^–5^ times. The samples were subsequently coated with a premixed fungal, actinomycetes, and bacterial media, sealed with a sealing film and cultured in an incubator at 28 °C. Microbial colonies were counted when they appeared on the medium until the colonies grew into blurred patches. The microorganisms were expressed as clone numbers per gram of sample.

$${\text{Clones of culturable soil microorganisms}}\;({\text{cfu}} {\text{g}}^{{ - {1}}} ) = {\text{M}} \times {\text{D}}/({\text{m}}({1} - {\text{R}}))$$

where M is the microbial colony, D is the dilution times, m is the weight of the fresh soil sample (g) and R is the soil relative water content (%).

The functional diversity of the soil microbial communities was determined according to Zak et al.^35^. After incubation, the data on a Biolog microplate reader were read every 24 h (1 day) for 8 readings (8 days). The metabolic capacity of soil microorganisms to metabolize 31 carbon sources was reflected by the average rate of colour change (AWCD) on Biolog microtiter plates.

$${\text{AWCD}}=\Sigma \left( {{\text{Ci}} - {\text{R}}} \right)/{31}$$

where Ci is the optical density at 590 nm for each reaction hole, R is the optical density values of ecological board control holes (dd-water) and holes with a Ci-R less than 0 are all noted as 0 in the calculations.

The culture samples obtained after 5 days of vigorous microbial growth and metabolism were selected for functional diversity analysis of microbial communities. The numbers of coloured holes were used to express the intensity of carbon source utilization, and the Shannon–Wiener diversity index (Hʹ), Evenness index (E), Simpson diversity index (D), and McIntosh index (U) were calculated^36,37^. The distributions of carbon sources in the Biolog EcoPlate are shown in Table S1.

$$\begin{gathered} H^{\prime} = -\sum {{\text{PilnPi}}} \hfill \\ E = H^{\prime}/{\text{InS}} \hfill \\ D = 1 - \sum {{\text{Pi}}^{2} } \hfill \\ U = \sqrt {\left( {\sum {{\text{ni}}^{2} } } \right)} \hfill \\ \end{gathered}$$

where Pi is the relative absorbance value of the i-th hole/sum of all absorbances and S is the absorbance value for 31 carbon sources that can be utilized by the microbes.

### Emergence rate of Codonopsis seedlings

Three 400 cm^2^ (20 cm × 20 cm) sample quadrants in the centre of each plot were designated for the investigation of the number of plants that emerged per m^2^ (Qs). Finally, the emergence rate (Er) was calculated according to the seeds sown per m^2^ (m_2_, g) and 1000-seed weight (m_1_) using the following equation:

$${\text{Er}} = \left( {{\text{m}}_{{1}} \times {\text{Qs}}} \right)/\left( {{\text{m}}_{{2}} \times {1}000} \right) \times {1}00\%$$

### Root viability of ***Codonopsis*** seedlings

The root viability of *Codonopsis* seedlings was determined according to a slightly modified 2,3,5-triphenyl tetrazolium chloride (TTC) reduction method from Monika Dalal and Renu Khanna-Chopra^38^. Briefly, 0.05 g of detached roots were incubated in 3 mL of 0.08% TTC in phosphate buffer (pH 7.5) for 24 h in the dark at 18 °C. Then, the samples were collected and washed with distilled water 3 times. The reduced TTC in fresh roots was extracted with 5 mL of 95% ethanol at 60 °C in the dark for 10 min, and the reduction in TTC, which represents root viability, was determined by determining the absorbance at 485 nm (UV 2450/Vis spectrometer).

$${\text{Root viability}}\left( {{\text{OD}}_{{{\text{485nm}}}} {\text{g}}^{{ - {1}}} } \right) = {\text{OD}}_{{{\text{485nm}}}}$$

### Assays of lipid peroxidation

Lipid peroxidation was measured following treatment and after 5 h by using malondialdehyde (MDA) equivalents^39^. The automatic rate (AR) was calculated as the average increase in the MDA concentration per hour^40^.

$${\text{AR}} = \left( {{\text{5 h MDA}} - 0{\text{ h MDA}}} \right)/{5}$$

### Assays of the antioxidant enzymatic activity

The antioxidant enzymatic activity was measured at 4 °C. The samples were homogenized in liquid nitrogen with 5 mL of extraction buffer containing 0.2 mM ethylenediaminetetraacetic (EDTA) and 2% polyvinylpyrrolidone (PVP) in 25 mM PBS (pH 7.8). The homogenate was centrifuged at 15,000 r min^−1^ for 20 min. The supernatant was subjected to antioxidant enzyme analysis^41–43^.

The activity of SOD was expressed as U g^−1^, and the CAT and POD activities were expressed as U g^−1^ min^−1^ according to Niu et al*.*^43^.

### Re-green rate of transplanted ***C. pilosula***

After transplanting, the dynamics of the seedlings that re-greened in each plot were periodically observed and recorded, and the re-green rate was calculated as follows:

$${\text{Re - green rate }}\left( \% \right) = {\text{number of turning green plants}}/{\text{total seedlings transplanted}} \times {1}00\%$$

### Determination of the quality and yield of ***Codonopsis*** seedlings and ***Codonopsis*** Radix

After harvesting on 15 Mar. 2020, the yield and incidence of disease in *Codonopsis* seedlings in each plot were measured. When 2-year-old *C. pilosula* was harvested on 10 Nov. 2020, the yield and incidence of root rot in each plot were measured. A total of 20 plants were randomly sampled from each plot to determine the quality of the roots, such as length, taproot length, taproot diameter, fresh weight per root, and lateral root number. After drying in the shade, the dry weight of a single root was measured, and the drying rate was calculated based on the fresh and dried weight.

$$\mathrm{Incidence of root rot }\left(\mathrm{\%}\right)=\mathrm{numbers of diseased roots}/\mathrm{total investigated roots}$$

Root length and taproot length were measured with a measuring tape (cm, 1/10); the taproot diameter was measured with a Vernier caliper (mm, 1/100); and the fresh and dry weight of a single roots was measured with an electronic scale (g, 1/100).

Subsequently, the water content, total ash content, acid-insoluble ash content, and extract content were measured according to the methods of Chinese Pharmacopoeia^3^. The polysaccharide content was measured via the phenol sulfuric acid method in SN/T 4260-2015^44^. Lobetyolin was measured using the assay of Yang et al*.*^45^ and Gao et al*.*^46^.

### Evaluation of ***Codonopsis*** Radix safety

Residues of methyl isothiocyanate, a metabolite of DZ, were analysed by GC‒MS^47^. Briefly, 10.0 g of a 2-year-old *C. pilosula* root sample was accurately weighed and mixed with 10 mL of ethyl acetate in a 50 mL centrifuge tube. After vortexing for 2 min, 4.0 g of NaCl was added, and the mixture was shaken well. After centrifugation at 4000 r min^−1^ for 5 min, 1 mL of the supernatant was taken and passed to a 0.22 μm microporous membrane for GC‒MS analysis with an OB-1701 MS capillary column (60 m × 250 μm × 0.25 μm). The analysis conditions consisted of a He carrier gas, 1 mL min^−1^ column flow, 1 μL injection volume, no diversion, an injection port temperature of 150 °C, and a gas mass interface temperature at of 180 °C. When the column temperature was increased, the initial temperature was set to 50 °C for 4 min and increased to 150 °C at 20 °C min^−1^ over 2 min. The retention time of the peak for the target compound was 8.9 min.

### Statistical analysis

Analyses of variance (ANOVAs) and principal component analysis (PCA) were conducted using SPSS 22.0 software (USA), and the least significant difference (LSD) method at *P* = 0.05 (LSD 0.05) was used for multiple comparisons.

A comprehensive evaluation was conducted according to the methods of Jin et al.^48^. First, based on factor analysis, the principal component values of each indicator whose initial characteristic root was greater than 1 were extracted, and then, the weight (*W*_*j*_) and positive and negative membership function values were calculated according to the relevant properties. Finally, the comprehensive index (*CI*) was estimated.

$$\begin{aligned} & W_{j} = \sum {\left( {C_{{l,j}} \times VP_{l} } \right)} /\sum {\sum {\left( {C_{{l,j}} \times VP_{l} } \right)} } \\ & R\left( {X_{{ij}} } \right) = (X_{{ij}} - X_{{j{\text{min}}}} )/(X_{{j{\text{max}}}} - X_{{j{\text{min}}}} ) \\ & RR\left( {X_{{ij}} } \right) = 1 - (X_{{ij}} - X_{{j{\text{min}}}} )/(X_{{j{\text{max}}}} - X_{{j{\text{min}}}} ) \\ & CI_{j} = \sum {\left[ {R\left( {X_{{ij}} } \right) \times W_{j} } \right]} \\ \end{aligned}$$

where *C*_*l,j*_ represents the l-th principal component of the j-th indicator, *VP*_*l*_ is the percentage of the variance of the first principal component, *W*_*j*_ is the weight value of the j-th indicator, i is different treatment, j is the measurement indicator, *R(X*_*ij*_*)* is the membership function value of indicator j under i treatment, *RR(X*_*ij*_*)* represents the value of anti membership function of i processing j indicator, *X*_*ij*_ is the average observation value of indicator j of i treatment, *X*_*jmin*_ is the minimum value of j indicator in all treatments, *X*_*jmax*_ is the maximum value of j indicator in all treatments, *CI*_*j*_ is the cumulative composite indicator of the i-th indicator. *CI*_*j*_ is the cumulative composite indicator of the i-th processed j indicator.

## Results

### Effect of DZ soil fumigation on soil enzymatic activity

**Figure 1:**
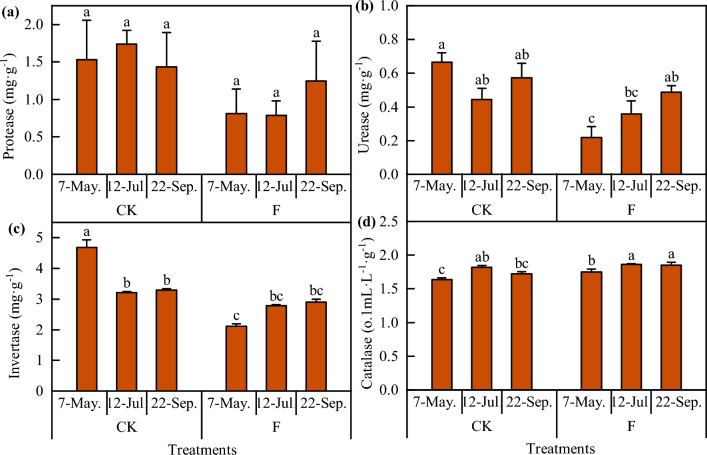
Effect of DZ soil fumigation on soil enzymatic activity. F means soil fumigation, and CK means soil non-fumigation. The data in the figure is the X ± SD. Different small letters mean a significant difference at *P* < 0.05.

Soil enzymes are involved in plant growth activities and play a prominent role in soil nutrient cycling and plant nutrient supply. Soil microorganisms and plant root secretions are significant sources of soil enzymes. As shown in Fig. 1, soil fumigation significantly decreased soil urease, protease, and invertase activity but not catalase activity before sowing. With the growth of *Codonopsis* seedlings, soil urease, protease, and cellulase activity began to increase, and there were no significant changes compared with those in the CK treatment. However, after soil fumigation, the soil catalase activity was greater than that in the CK treatment.

### Effect of DZ fumigation on the number of culturable soil microorganisms

**Figure 2:**
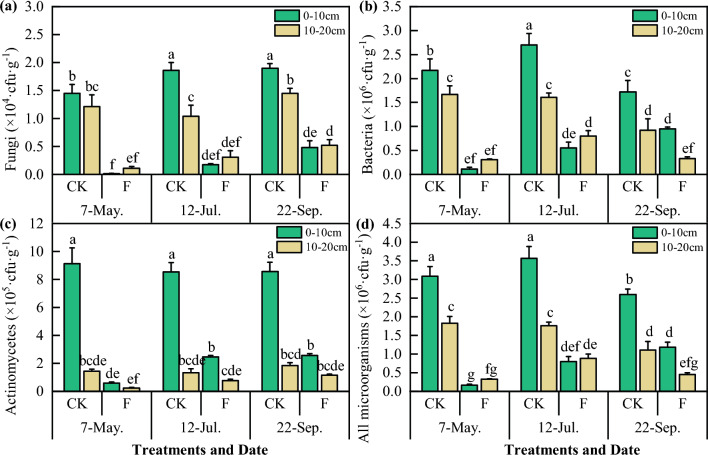
Effect of DZ fumigation on the culturable soil microbial quantity in *Codonopsis* seedlings fields. F means soil fumigation, and CK means soil non-fumigation. The data in the figure is the X ± SD. Different small letters mean a significant difference at *P* < 0.05.

Culturable soil microorganisms play a crucial role in the decomposition, metabolism, and other processes of substances in soils. Throughout the reproductive period of *Codonopsis* seedlings, the number of colonies of culturable microbes in the soil increased in the order bacteria > actinomycetes > fungi, and actinomycetes were most affected by soil fumigation but recovered more easily than fungi and bacteria (Fig. 2). The number of culturable soil microorganisms in the 10–20 cm soil layer was lower than that in the 0–10 cm layer. Compared with that in the CK treatment, the number of fungi in the fumigated block decreased by 95.72% on 7 May, 83.53% on 12 Jul. and 70.19% on 22 Sep.; the number of bacteria decreased by 89.17% on 7 May, 68.55% on 12 Jul. and 51.38% on 22 Sep.; and the number of actinomycetes decreased by 92.35% on 7 May, 67.20% on 12 Jul., and 64.32% on 22 Sep..

### Effect of DZ soil fumigation on the metabolic functional diversity of soil microbial communities

**Figure 3:**
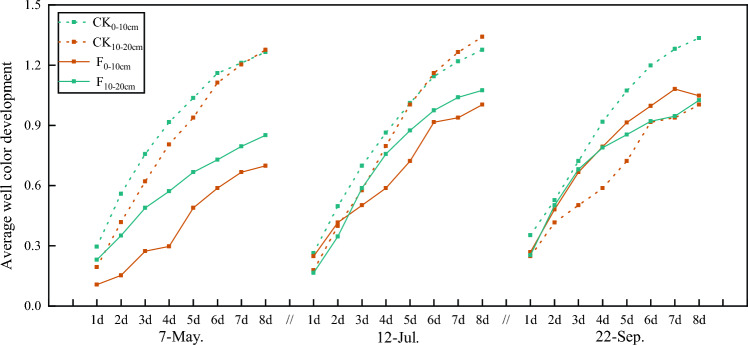
AWCD of soil microbial community under different treatments. F means soil fumigation, and CK means soil non-fumigation. The data in the figure is the X ± SD.

AWCD is a crucial indicator of the metabolism of soil microbial carbon sources and can reflect the ability of soil microbial communities to utilize carbon sources. The faster the AWCD value increases, the more the microbial community metabolizes the carbon source and vice versa. The AWCD values of each soil layer rapidly increased from 1 to 5 days, and after 5 days, the increase in AWCD values slowed. This indicates that the microbial community strongly metabolized carbon sources from 1 to 5 days, while after 5 days, the microbial community metabolism of carbon sources gradually weakened. Overall, soil microbial activity was weakest in the fumigation plot before sowing, where soil microbial activity was more strongly affected in the 0–10 cm soil layer than in the 10–20 cm layer (Fig. 3).

**Figure 4:**
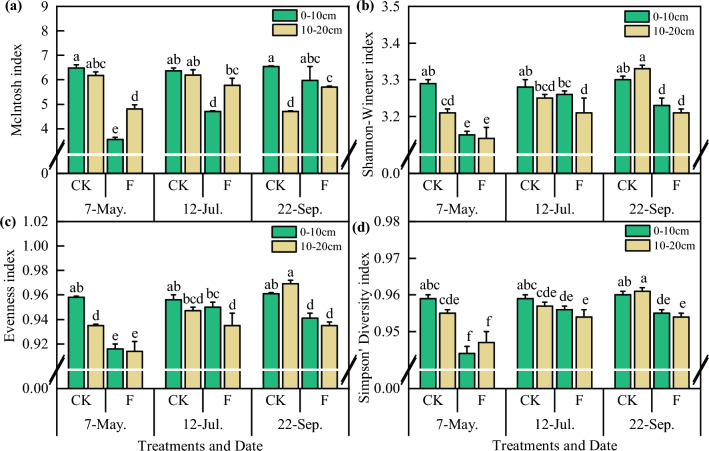
Soil microbial diversity index of *Codonopsis* seedlings field with different treatments. F means soil fumigation, and CK means soil non-fumigation. The data in the figure is the X ± SD.

As shown in Fig. 4, soil fumigation reduced the microbial diversity index and Evenness index of each soil layer before sowing, which increased with the growth of *Codonopsis* seedlings. Among the indices, the McIntosh indices were all above 1, which also indicated that there were only a few microbial species in the communities. The Evenness index varied between 0 and 1.0, and the variation in the Evenness index was consistent with that of the Shannon–Wiener index.

**Figure 5:**
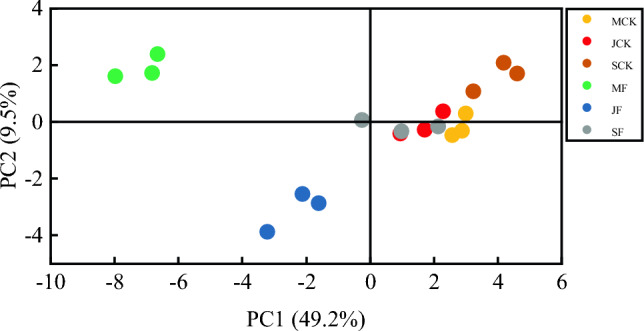
PCA of carbon utilization of soil microbes under different dates of different treatments. F means soil fumigation, CK means soil non-fumigation, M—May, J—July, and S—September.

To visualize the effect of dazomet fumigation on the metabolic characteristics of soil microbial communities, principal component analysis was carried out in this study using AWCD values from 5 days of incubation (Fig. 5). Soil fumigation affected soil microbial carbon source utilization in the early and middle stages of *Codonopsis* seedlings development, the effects gradually disappeared in the later stages, and the differences were not significant.

### Effect of DZ soil fumigation on the emergence rate of ***Codonopsis*** seedlings

**Figure 6:**
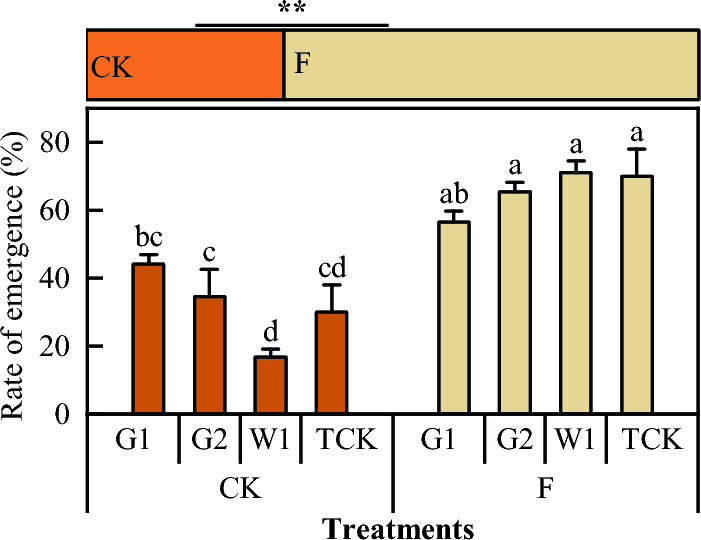
The emergence rate of *Codonopsis* seedlings. Data in the figure is the X ± SD. Different small letters mean a significant difference at *P* < 0.05; *is a significant correlation at *P* < 0.05.

Soil fumigation had significant effects on the emergence rate of *Codonopsis* seedlings (F = 27.49, *P* < 0.05; Fig. 6) relative to non-fumigated soil. The average emergence rates of the four cultivars of *C. pilosula* in the fumigation plot (F) and the non-fumigated plot (CK) were 65.75% and 31.38%, respectively. The emergence rate of the F plot was 34.38% greater than that of the CK plot. The seedling emergence rate in W1 increased by 322.3% compared with that in the corresponding control, showing the most significant increase, and that in G1 increased by 22.2%, the smallest increase.

### Effect of DZ soil fumigation on physiological characteristics of ***Codonopsis*** seedlings

**Figure 7:**
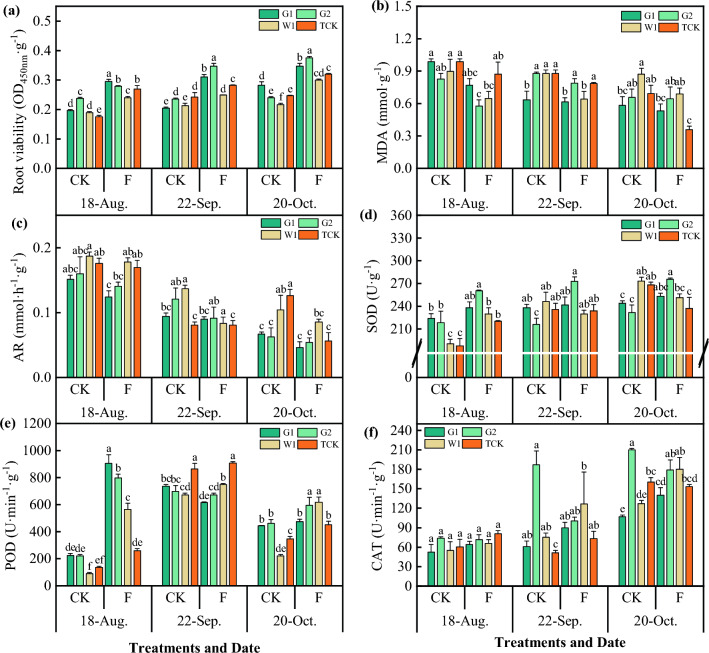
The physiological characteristics of *Codonopsis* seedlings. Data in the figure is the X ± SD. Different small letters mean a significant difference at *P* < 0.05.

As shown in Fig. 7, soil fumigation had a significant effect on the physiological characteristics of the roots of *Codonopsis* seedlings (*P* < 0.05). Soil fumigation had a significant effect on the viability of the roots of *Codonopsis* seedlings of each cultivar, and the effect increased gradually with the growing period. Root viability of the seedlings under soil fumigation was 34.87% greater than that under CK treatment during the three growing periods. The degree of membrane lipid peroxidation is indicated by the content of MDA. The MDA and AR contents in *Codonopsis* seedling roots decreased gradually with the growing period, and the MDA content in the F plot was significantly lower than that in the CK plot (*P* < 0.05). Compared with that in CK, the average MDA content in the F plot decreased by 19.07%, and the AR decreased by 18.28%. In August, the effects of fumigation on the MDA content in G1, G2, and W1 were significant, but those on the TCK content were not significant. In September and October, W1 was significantly affected. The results showed that soil fumigation could reduce the content of MDA in *Codonopsis* seedlings and thus reduce cell membrane lipid damage.

Moreover, the soil fumigation had a significant effect on the POD activity of *Codonopsis* seedlings. Compared with that in the CK plot, the POD activity increased by 336.83% in August (*P* < 0.05). Soil fumigation also had a significant effect on the SOD activity of *Codonopsis* seedlings (*P* < 0.05) in August. However, there was no significant difference in CAT activity in the different growth stages (*P* > 0.05).

### Effect of DZ soil fumigation on the yield and root rot incidence rate of ***Codonopsis*** seedlings

**Figure 8:**
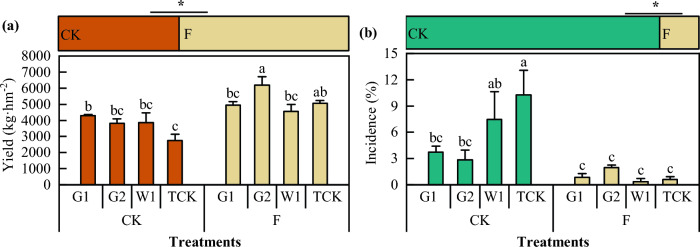
The yield and incidence rate of *Codonopsis* seedlings. Data in the figure is the X ± SD. Different small letters mean a significant difference at *P* < 0.05; “*” is a significant correlation at *P* < 0.05.

As shown in Fig. 8, there was a significant difference in the yield of *Codonopsis* seedlings among the cultivars. The yield of the F plot was greater than that of CK. The yield of G2 in the F plot was the highest, at approximately 6182.00 kg hm^−2^. The yield of W1 was the lowest, at approximately 4554.53 kg hm^−2^. The yield of G1 in the CK plot was the highest, at approximately 429.90 kg hm^−2^. The yield of the WCK treatment was the lowest, at approximately 2750.80 kg hm^−2^. The yield in the F plot was 1543.23 kg hm^−2^ greater than that in the CK plot (42.4%), and the difference between them was significant (*P* < 0.05). Soil fumigation had the most significant effect on G2, with a yield increase of 2362.07 kg hm^−2^ (increase of 61.8%), while soil fumigation had the weakest effect on G1, with a yield increase of 646.60 kg hm^−2^ (increase of 15.4%). Moreover, soil fumigation significantly affected the incidence of root rot in *Codonopsis* seedlings. Compared with that in the CK treatment, the incidence of root rot in the F treatment decreased by 83.9%.

### Re-green rate and survival rate of ***C. pilosula*** after transplantation

**Figure 9:**
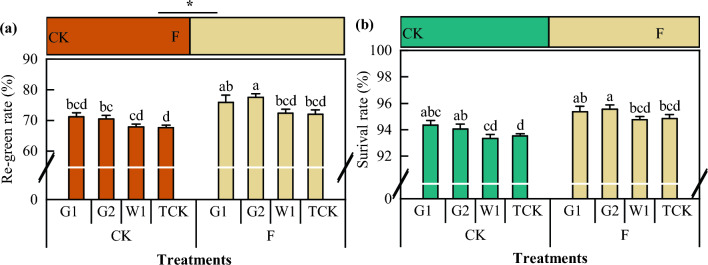
The re-green rate and survival rate of *C. pilosula.* Data in the figure is the X ± SD. Different small letters mean a significant difference at *P* < 0.05; “*” is a significant correlation at *P* < 0.05.

After *Codonopsis* seedlings were transplanted and turned green, the individual phenological distributions of the different cultivars of *C. pilosula* changed to varying degrees (Fig. 9). Compared with that in the CK treatment, the re-green rate in the F treatment increased by 10.09%, and the survival rate increased by 1.62%. Among them, the re-green rate in G2 was significantly greater than that in W1 and TCK (*P* < 0.05).

### Effects of seedlings on the yield and quality of ***Codonopsis*** Radix

**Figure 10:**
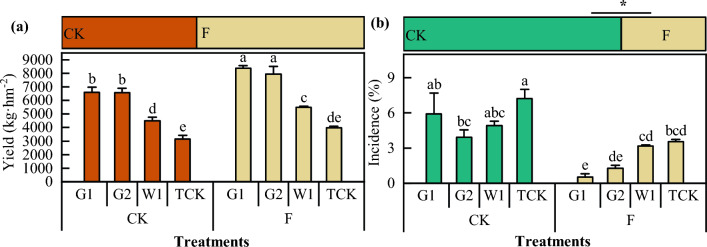
The yield and incidence of *Codonopsis* Radix. Data in the figure is the X ± SD. Different small letters mean a significant difference at *P* < 0.05; “*” is a significant correlation at *P* < 0.05.

When different treatment *Codonopsis* seedlings were cultivated, the yield of *Codonopsis* Radix increased (*P* < 0.05), and root rot incidence of *Codonopsis* Radix decreased (Fig. 10). Compared with that of CK, the yield of *Codonopsis* Radix increased by 23.94%, and the root rot incidence decreased by 61.32%. There were also differences among the different cultivars of *C. pilosula*. For instance, the yields of G1, G2, W1, and TCK increased by 26.51%, 20.99%, 22.57%, and 26.68%, respectively, and the root rot incidence decreased by 91.06%, 67.43%, 35.44%, and 51.18%, respectively.

**Figure 11:**
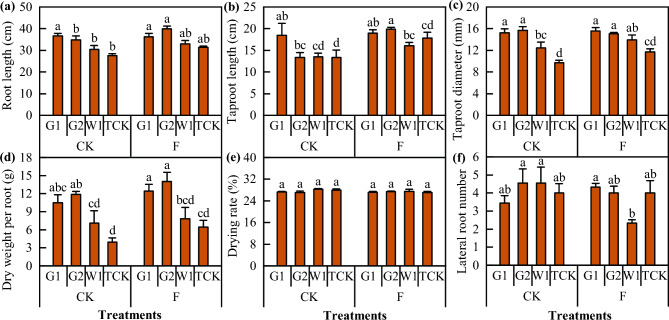
The external quality of *Codonopsis* Radix. Data in the figure is the X ± SD. Different small letters mean a significant difference at *P* < 0.05.

**Figure 12:**
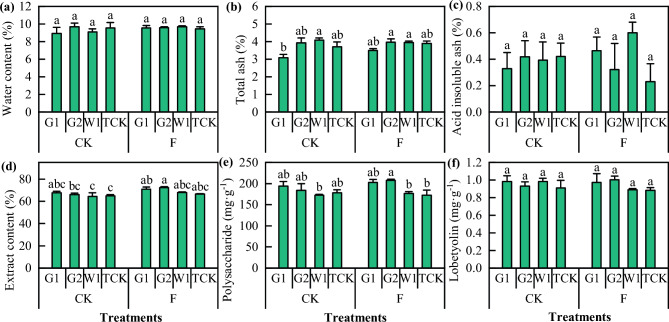
The internal component content of *Codonopsis* Radix. Data in the figure is the X ± SD. Different small letters mean a significant difference at *P* < 0.05.

The differences in the external quality (Fig. 11) and internal component content (Fig. 12) of *Codonopsis* Radix were also determined. The transplanted *Codonopsis* seedlings cultivated after soil fumigation exhibited improvements in terms of root length, taproot root length, taproot root diameter, and single dry weight of *Codonopsis* Radix to varying degrees, and the most significant effect was observed in G2 (*P* < 0.05). However, this treatment had no significant effect on the drying rate or the number of lateral roots of *Codonopsis* Radix. Moreover, there was no significant effect on the internal component content of *Codonopsis* Radix, although the extract composition of the different cultivars was different.

### Comprehensive factor analysis of different ***C. pilosula*** cultivars under different treatments

Based on principal component analysis of thirty-eight indicators for four cultivars of *Codonopsis* seedlings and *Codonopsis* Radix under the two treatments, the characteristic roots of the first six principal components were greater than 1. The contribution rates were 49.83%, 15.56%, 11.63%, 9.56%, 7.39%, and 3.47%, respectively. The cumulative contribution rate was 97.44%. Therefore, the characteristic roots and contribution rates of the first six principal components (Table S2) were extracted to calculate the weight values of each indicator (Table S3). According to the test indicator membership degree, weight value, and addition and multiplication of all the indicators of *Codonopsis* seedlings and *Codonopsis* Radix, the comprehensive evaluation indices were F > CK (Table S4), and in the CK treatment, the order was G1 > G2 > W1 > TCK, in F was G2 > G1 > W1 > TCK.

### Safety evaluation of ***Codonopsis*** Radix

*Codonopsis* Radix is a traditional Chinese medicine used for medicine and food. Currently, agencies in China have set standards for the maximum residual content of methyl isothiocyanate allowable in food, but the threshold for the residual content of methyl isothiocyanate in food specified by the EU is 0.02 mg kg^−1^. In this study, the content of methyl isothiocyanate residues in *Codonopsis* Radix was determined. Methyl isothiocyanate was not detected in *Codonopsis* Radix (< 0.02 mg kg^−1^).

## Discussion

### *Codonopsis* seedling emergence rate and root viability can be enhanced by dazomet soil fumigation

Root systems are essential functional organs in plants that absorb nutrients and water, participate in the process of biosynthesis and transformation, and have a vital impact on the growth and yield of crops^49,50^. Soils are essential for material and energy exchange, and a soil’s microecological environment directly affects the growth and development of plants. Numerous studies have shown that the application of soil amendments is an effective way to improve the soil microecological environment and increase crop quality and yield^51–53^. In our study, we found that, during the pre-seedling period, DZ soil fumigation affected soil enzymes, the number of culturable soil microorganisms, and the functional diversity of the microbial community; however, all the microbial communities recovered to varying degrees during the post-seedling period. Thus, due to the altered soil environment, fumigation also had an impact on the emergence and root viability of *Codonopsis* seedlings. The emergence rate of *Codonopsis* seedlings increased by 34.38% and root viability increased by 38.42% compared with CK, among which the root viability of TCK was most responsive and G2 was least responsive to the environment. This difference may be due to the strong adaptability of TCK, a local cultivar, to continuous cropping, while G2 was more environmentally adaptable. Liu et al.^19^ studied the effect of dazomet on the growth of Pingyi sweet tea seedlings in pots and reported that dazomet increased the root viability of Pingyi sweet tea by 48.8%, which was similar to the findings of the present study.

### Soil fumigation with DZ improved the environmental adaptability of ***Codonopsis*** seedlings

MDA is the final product of membrane lipid peroxidation. A high concentration of MDA has a toxic effect on plants, but a low concentration of MDA can induce enhanced stress tolerance^41,42^. The rate of tissue automatic oxidation (AR) is the rate of MDA production per unit of time and can directly reflect the degree of membrane lipid peroxidation^41,42,54^. Under continuous cropping conditions, the abundance of weeds^55^ and harmful bacteria^56^ in the soil increases, which might lead to stress during plant growth. Under adverse conditions, large quantities of reactive oxygen species such as hydrogen peroxide, superoxide anions, and hydroxyl radicals are produced in plants, leading to an increase in MDA content, which in turn affects plant growth^57^. To scavenge reactive oxygen species, plants activate an antioxidant defence enzyme system consisting of enzymes such as SOD, CAT, and POD^58,59^ and regulate dysregulated reactive oxygen species metabolism via protective enzyme activity in plants. Soil fumigation with dazomet can significantly reduce the number of weeds^55^ and the number of pathogenic bacteria^60^ in the soil. Although soil enzymatic activity and the functional diversity of the soil microbial community were reduced for a short duration, the microbial communities recovered to varying degrees during the later stages, and the *Codonopsis* seedlings were in a better soil environment with a lower MDA content and greater enzymatic activity. The high re-green rate and survival rate of *Codonopsis* seedlings further explained this difference. Therefore, it is evident that changes in the soil environment significantly improve the environmental adaptability of *Codonopsis* seedlings, allow for rapid regreening, and increase field productivity.

### Soil fumigation with DZ improved the quality and yield of ***Codonopsis*** seedlings

The appearance of *Codonopsis* seedlings is directly related to the quality of the medicine and its clinical efficacy. The quality of *Codonopsis* seedlings is determined by the genetic composition of the *C. pilosula* seeds, the growth environment and cultivation measures; therefore, the quality of *Codonopsis* seedlings can be improved under suitable soil environments and cultivation conditions^61^. Studies have shown that the application of soil amendments could improve the soil environment and increase crop yield and quality^62,63^. In this study, when soil moisture conditions and the amount, duration, and mulching material of fumigants were strictly regulated when dazomet was applied, soil fumigation increased the yield of *Codonopsis* seedlings by 42.4% and reduced the morbidity rate by 84.48%. Similarly, compared with the CK treatment, the yield of *Codonopsis* Radix increased by 23.94%, and the root rot incidence decreased by 61.32%. The effect of dazomet fumigation on the quality and yield components of *Codonopsis* seedlings was mainly reflected as the indicators of root length, root thickness, single root weight, and the number of lateral roots. This was further corroborated by the appearance of the *Codonopsis* Radix, showing that the quality of the seedlings was directly related to the growth and development of the plants after transplanting and the yield and quality of the herbs harvested in the same year^64^. Considering the seedling traits, root rot incidence, and yield, fumigation had a significant effect on the quality and yield of all the *C. pilosula* cultivars. The most significant effect was seen in G2, with a 61.8% increase in yield, and the weakest effect was observed in G1, with a 15.4% increase in yield. In addition, soil amendments had little effect on the intrinsic quality of *Codonopsis* Radix, which is generally determined by genetic factors, and the intrinsic quality varies significantly among cultivars. In this study, there were no significant differences in the ash, leachate content, polysaccharide, or lobetyolin content of *Codonopsis* Radix between the treatments; however, the polysaccharide and lobetyolin content of *Codonopsis* Radix varied among cultivars. Moreover, we did not find methyl isothiocyanate residues in *Codonopsis* Radix, indicating that the safety of *Codonopsis* Radix was high after fumigation of the seedbed by dazomet.

## Conclusions

Soil fumigation has been proven to be a critical technology for ensuring stable crop yields. The results of this study revealed that the application of dazomet could effectively improve the seeds’ emergence rate and seedling antioxidant enzymatic activity, and reduce membrane lipid peroxidation and the incidence of root rot. After transplant, the seedling re-green rate was increased, and the yield and appearance quality of *Codonopsis* Radix improved. These findings were closely related to those for *C. pilosula*, especially because of its high yield but poor resistance. Therefore, when herbs are bred that require seedling transplants, especially cultivars with good quality but poor resistance or species with rare germplasm resources, soil fumigation can be carried out before seedling, which not only improves yield but also, more importantly, ensures the probability of successful cultivation of the species.

### Supplementary Information

Supplementary Information.

## Data Availability

The data used to support the findings of this study are available from the corresponding author upon request.
