# Supplementary material for: Improvement in the quality and productivity of Codonopsis pilosula seedlings by dazomet soil fumigation
Source: Sci Rep. 2024 Mar 5;14:5407. doi: 10.1038/s41598-024-56093-3 (PMC10915150; doi:10.1038/s41598-024-56093-3)
Supplement: Supplementary file 1 — Supplementary Information. [file 41598_2024_56093_MOESM1_ESM.docx]

**Improvement in the quality and productivity of Codonopsis pilosula seedlings by dazomet soil fumigation**

Hongyan Wang, Yuan Chen^*^, Fengxia Guo^*^, Pengbin Dong, Wei Liang, Jiali Cheng

College of Agronomy, College of Life Science and Technology, State Key Laboratory of Aridland Crop Science, Gansu Agricultural University, Lanzhou 730070, China

*Correspondence:

Yuan Chen, Fengxia Guo

Agronomy College

Gansu Agricultural University

Lanzhou 730070, Gansu, China.

Emails: [chenyuan@gsau.edu.cn](mailto:chenyuan@gsau.edu.cn), guofx@gsau.edu.cn;

**Supplementary Table S1–S4**

**Table S1.** Biolog EcoPlate carbon source distribution.

**Table S2.** The result of principal components analysis of all indicators of *C. pilosula* seedlings and *Codonopsis* radix.

**Table S3.** Capacity and weight of all indicators of *C. pilosula* seedlings and *Codonopsis* radix.

**Table S4.** Membership value and comprehensive evaluation index of all indicators of *C. pilosula* seedlings and *Codonopsis* radix.

**Table S1.** Biolog EcoPlate carbon source distribution

| Carbon source distribution | Carbon source type | | |
| --- | --- | --- | --- |
| B2 | Saccharides | D-Xylose |  |
| H1 |  | α-D-Lactose |  |
| A2 |  | β-Methyl-D-Glucoside |  |
| G2 |  | α-D-Glucose-1-Phosphate |  |
| E1 |  | α-Cyclodextrin |  |
| F1 |  | Glycogen |  |
| G1 |  | D-Cellobiose |  |
| A4 | Amino acids | L-Arginine |  |
| B4 |  | L-Asparagine |  |
| C4 |  | L-Phenylalanine |  |
| D4 |  | L-Serine |  |
| E4 |  | L-Threonine |  |
| F4 |  | Glycyl-L-Glutamic Acid |  |
| B1 | Carboxylic acids | Pyruvic Acid Methyl Ester |  |
| C1 |  | Tween40 |  |
| D1 |  | Tween 80 |  |
| A3 |  | D-Galactonic Acid γ-Lactone |  |
| C2 | Alcohols | i-Erythritol |  |
| D2 |  | D-Mannitol |  |
| H2 |  | D,L-α-Glycerol Phosphate |  |
| G4 | Amine | Phenylethylamine |  |
| H4 |  | Putrescine |  |
| E2 |  | N-Acetyl-D-Glucosamine |  |
| B3 | Acids | D-Galacturonic Acid |  |
| F2 |  | D-Glucosaminic Acid |  |
| C3 |  | 2-Hydroxy Benzoic Acid |  |
| D3 |  | 4-Hydroxy Benzoic Acid |  |
| E3 |  | γ-Hydroxybutyric Acid |  |
| F3 |  | Itaconic Acid |  |
| G3 |  | α-Ketobutyric Acid |  |
| H3 |  | D-Malic Acid |  |

**Table S2.** The result of principal components analysis of all indicators of *C. pilosula* seedlings and *Codonopsis* radix

| Principal components | Eigenvalue | Contribution rate (%) | Cumulative contribution rate (%) |
| --- | --- | --- | --- |
| 1 | 18.934 | 49.827 | 49.827 |
| 2 | 5.913 | 15.56 | 65.387 |
| 3 | 4.418 | 11.627 | 77.013 |
| 4 | 3.634 | 9.563 | 86.577 |
| 5 | 2.808 | 7.39 | 93.967 |
| 6 | 1.32 | 3.473 | 97.440 |

**Table S3.** Capacity and weight of all indicators of *C. pilosula* seedlings and *Codonopsis radix*

| Indicators | | | load | | | | | | Weight value |
| --- | --- | --- | --- | --- | --- | --- | --- | --- | --- |
|  |  |  | 1 | 2 | 3 | 4 | 5 | 6 |  |
| *Codonopsis* seedlings | Emergence | | 0.744 | -0.55 | 0.264 | 0.143 | -0.148 | 0.173 | 0.029 |
|  | TTC | 18-Aug | 0.89 | -0.26 | -0.093 | 0.050 | 0.207 | 0.016 | 0.020 |
|  |  | 22-Sep | 0.787 | -0.134 | -0.013 | 0.491 | 0.275 | -0.198 | 0.029 |
|  |  | 20-Oct | 0.904 | -0.127 | 0.295 | 0.277 | 0.042 | -0.044 | 0.023 |
|  | MDA | 18-Aug | 0.743 | -0.148 | -0.414 | 0.421 | -0.201 | 0.187 | 0.032 |
|  |  | 22-Sep | 0.579 | 0.019 | 0.458 | -0.246 | -0.579 | 0.087 | 0.031 |
|  |  | 20-Oct | 0.473 | -0.529 | 0.524 | -0.296 | 0.345 | -0.089 | 0.035 |
|  | AR | 18-Aug | 0.822 | 0.289 | 0.147 | -0.199 | 0.106 | -0.401 | 0.029 |
|  |  | 22-Sep | 0.337 | -0.534 | 0.613 | 0.218 | -0.221 | -0.337 | 0.036 |
|  |  | 20-Oct | 0.870 | -0.048 | 0.028 | -0.377 | 0.267 | 0.138 | 0.025 |
|  | POD | 18-Aug | -0.892 | -0.046 | 0.037 | -0.266 | 0.212 | 0.174 | 0.023 |
|  |  | 22-Sep | -0.458 | -0.689 | 0.457 | 0.093 | 0.202 | -0.021 | 0.028 |
|  |  | 20-Oct | 0.785 | -0.404 | -0.048 | 0.035 | -0.354 | 0.003 | 0.022 |
|  | SOD | 18-Aug | 0.979 | -0.010 | -0.007 | 0.042 | -0.052 | 0.081 | 0.014 |
|  |  | 22-Sep | 0.420 | 0.493 | 0.198 | 0.684 | 0.185 | 0.124 | 0.033 |
|  |  | 20-Oct | -0.114 | 0.511 | -0.003 | 0.847 | -0.068 | -0.055 | 0.026 |
|  | CAT | 18-Aug | 0.416 | -0.727 | -0.243 | 0.043 | 0.484 | 0.035 | 0.029 |
|  |  | 22-Sep | 0.277 | -0.255 | -0.840 | -0.361 | -0.079 | 0.030 | 0.029 |
|  |  | 20-Oct | 0.198 | -0.533 | -0.737 | 0.097 | 0.038 | -0.217 | 0.028 |
|  | Yield | | 0.885 | 0.001 | 0.087 | 0.214 | 0.174 | 0.364 | 0.025 |
|  | Incidence rate | | 0.814 | -0.367 | -0.024 | -0.251 | -0.081 | 0.335 | 0.027 |
| *C*. *pilosula* | Rejuvenation | | 0.984 | 0.022 | 0.038 | 0.167 | 0.026 | -0.022 | 0.015 |
|  | Survival | | 0.963 | -0.167 | 0.171 | 0.119 | -0.002 | 0.008 | 0.017 |
| *Codonopsis* radix | Taproot length | | 0.841 | 0.099 | 0.512 | 0.06 | 0.045 | 0.118 | 0.023 |
|  | Root length | | 0.877 | 0.344 | -0.062 | -0.178 | -0.001 | 0.096 | 0.020 |
|  | Taproot diameter | | 0.724 | 0.331 | -0.299 | -0.482 | -0.169 | 0.111 | 0.031 |
|  | Fresh weight per root | | 0.829 | 0.382 | -0.282 | -0.252 | 0.082 | -0.031 | 0.025 |
|  | Dry weight per root | | 0.828 | 0.393 | -0.288 | -0.236 | 0.07 | -0.021 | 0.025 |
|  | Drying rate | | -0.662 | 0.418 | -0.117 | 0.574 | -0.182 | 0.087 | 0.030 |
|  | Lateral root number | | 0.127 | -0.382 | 0.267 | 0.087 | -0.794 | 0.261 | 0.033 |
|  | Yield | | 0.855 | 0.409 | -0.183 | -0.22 | -0.102 | -0.098 | 0.025 |
|  | Incidence | | 0.882 | -0.028 | -0.237 | 0.148 | 0.065 | 0.088 | 0.019 |
|  | Water content | | -0.322 | 0.63 | 0.519 | -0.235 | 0.058 | 0.414 | 0.034 |
|  | Total ash | | 0.127 | 0.331 | 0.664 | -0.525 | -0.23 | -0.313 | 0.037 |
|  | Acid insoluble ash | | 0.035 | 0.078 | 0.428 | -0.066 | 0.843 | 0.176 | 0.030 |
|  | Extract content | | 0.951 | 0.151 | 0.088 | 0.214 | -0.082 | -0.106 | 0.021 |
|  | Polysaccharide content | | 0.793 | 0.475 | 0.071 | 0.001 | 0.005 | -0.334 | 0.023 |
|  | Lobetyolin | | 0.327 | 0.929 | -0.031 | 0.06 | 0.115 | 0.045 | 0.021 |

**Table S4.** Membership value and comprehensive evaluation index of all indicators of *C. pilosula* seedlings and *Codonopsis* radix

| Indicators | | | CK | | | | F | | | |
| --- | --- | --- | --- | --- | --- | --- | --- | --- | --- | --- |
|  |  |  | G1 | G2 | W1 | TCK | G1 | G2 | W1 | TCK |
| *Codonopsis* seedlings | Emergence | | 0.502 | 0.326 | 0.000 | 0.244 | 0.731 | 0.897 | 1.000 | 0.978 |
|  | TTC | 18-Aug | 0.177 | 0.522 | 0.119 | 0.000 | 1.000 | 0.871 | 0.542 | 0.782 |
|  |  | 22-Sep | 0.000 | 0.215 | 0.060 | 0.261 | 0.740 | 1.000 | 0.316 | 0.541 |
|  |  | 20-Oct | 0.415 | 0.149 | 0.000 | 0.193 | 0.821 | 1.000 | 0.528 | 0.649 |
|  | MDA | 18-Aug | 0.000 | 0.387 | 0.218 | 0.000 | 0.530 | 1.000 | 0.827 | 0.284 |
|  |  | 22-Sep | 0.931 | 0.007 | 0.000 | 0.000 | 1.000 | 0.347 | 0.903 | 0.361 |
|  |  | 20-Oct | 0.565 | 0.422 | 0.000 | 0.349 | 0.664 | 0.448 | 0.359 | 1.000 |
|  | AR | 18-Aug | 0.574 | 0.432 | 0.000 | 0.182 | 1.000 | 0.740 | 0.148 | 0.286 |
|  |  | 22-Sep | 0.763 | 0.284 | 0.000 | 0.999 | 0.837 | 0.809 | 0.953 | 1.000 |
|  |  | 20-Oct | 0.736 | 0.794 | 0.270 | 0.000 | 1.000 | 0.899 | 0.504 | 0.872 |
|  | POD | 18-Aug | 0.837 | 0.840 | 1.000 | 0.944 | 0.000 | 0.131 | 0.417 | 0.793 |
|  |  | 22-Sep | 0.417 | 0.284 | 0.199 | 0.857 | 0.000 | 0.199 | 0.461 | 1.000 |
|  |  | 20-Oct | 0.561 | 0.602 | 0.000 | 0.316 | 0.636 | 0.938 | 1.000 | 0.580 |
|  | SOD | 18-Aug | 0.497 | 0.424 | 0.041 | 0.000 | 0.692 | 1.000 | 0.578 | 0.448 |
|  |  | 22-Sep | 0.383 | 0.000 | 0.533 | 0.344 | 0.453 | 1.000 | 0.243 | 0.314 |
|  |  | 20-Oct | 0.291 | 0.000 | 0.950 | 0.839 | 0.491 | 1.000 | 0.455 | 0.129 |
|  | CAT | 18-Aug | 0.000 | 0.748 | 0.082 | 0.274 | 0.405 | 0.674 | 0.471 | 1.000 |
|  |  | 22-Sep | 0.070 | 1.000 | 0.177 | 0.000 | 0.281 | 0.362 | 0.554 | 0.160 |
|  |  | 20-Oct | 0.000 | 1.000 | 0.199 | 0.521 | 0.325 | 0.699 | 0.713 | 0.452 |
|  | Yield | | 0.451 | 0.312 | 0.320 | 0.000 | 0.640 | 1.000 | 0.526 | 0.672 |
|  | Incidence rate | | 0.659 | 0.749 | 0.284 | 0.000 | 0.950 | 0.840 | 1.000 | 0.975 |
| *C*. *pilosula* | Rejuvenation | | 0.347 | 0.286 | 0.020 | 0.000 | 0.827 | 1.000 | 0.469 | 0.439 |
|  | Survival | | 0.455 | 0.318 | 0.000 | 0.091 | 0.909 | 1.000 | 0.636 | 0.682 |
| *Codonopsis* radix | Taproot length | | 0.786 | 0.000 | 0.029 | 0.003 | 0.866 | 1.000 | 0.413 | 0.689 |
|  | Root length | | 0.740 | 0.582 | 0.235 | 0.000 | 0.703 | 1.000 | 0.437 | 0.318 |
|  | Taproot diameter | | 0.923 | 1.000 | 0.463 | 0.000 | 0.980 | 0.899 | 0.708 | 0.332 |
|  | Fresh weight per root | | 0.656 | 0.799 | 0.302 | 0.000 | 0.851 | 1.000 | 0.375 | 0.259 |
|  | Dry weight per root | | 0.648 | 0.784 | 0.314 | 0.000 | 0.842 | 1.000 | 0.383 | 0.247 |
|  | Drying rate | | 0.201 | 0.051 | 1.000 | 0.740 | 0.115 | 0.335 | 0.395 | 0.000 |
|  | Lateral root number | | 0.500 | 0.000 | 0.000 | 0.250 | 0.100 | 0.250 | 1.000 | 0.250 |
|  | Yield | | 0.664 | 0.656 | 0.257 | 0.000 | 1.000 | 0.920 | 0.451 | 0.161 |
|  | Incidence | | 0.194 | 0.492 | 0.346 | 0.000 | 1.000 | 0.889 | 0.606 | 0.552 |
|  | Water content | | 1.000 | 0.000 | 0.802 | 0.194 | 0.212 | 0.164 | 0.027 | 0.316 |
|  | Total ash | | 1.000 | 0.152 | 0.000 | 0.373 | 0.579 | 0.121 | 0.126 | 0.179 |
|  | Acid insoluble ash | | 0.732 | 0.489 | 0.562 | 0.486 | 0.369 | 0.749 | 0.000 | 1.000 |
|  | Extract content | | 0.396 | 0.193 | 0.000 | 0.061 | 0.815 | 1.000 | 0.444 | 0.274 |
|  | Polysaccharide content | | 0.617 | 0.338 | 0.000 | 0.161 | 0.850 | 1.000 | 0.125 | 0.010 |
|  | Lobetyolin | | 0.824 | 0.392 | 0.817 | 0.221 | 0.723 | 1.000 | 0.072 | 0.000 |
| Comprehensive evaluation index | | | 0.522 | 0.411 | 0.258 | 0.257 | 0.634 | 0.733 | 0.505 | 0.502 |
| Comprehensive sorting | | | 3 | 6 | 7 | 8 | 2 | 1 | 4 | 5 |
